# Baricitinib ameliorates inflammatory and neuropathic pain in collagen antibody-induced arthritis mice by modulating the IL-6/JAK/STAT3 pathway and CSF-1 expression in dorsal root ganglion neurons

**DOI:** 10.1186/s13075-024-03354-1

**Published:** 2024-06-15

**Authors:** Kenta Makabe, Hiroyuki Okada, Naohiro Tachibana, Hisatoshi Ishikura, Norihito Ito, Masaru Tanaka, Ryota Chijimatsu, Asuka Terashima, Fumiko Yano, Meiko Asaka, Dai Yanagihara, Shuji Taketomi, Takumi Matsumoto, Sakae Tanaka, Yasunori Omata, Taku Saito

**Affiliations:** 1 Sensory and Motor System Medicine, Graduate School of Medicine, The University of Tokyo, Hongo 7-3-1, Bunkyo-Ku, Tokyo, 113-8655 Japan; 2 Bone and Cartilage Regenerative Medicine, Graduate School of Medicine, The University of Tokyo, 7-3-1 Hongo, Bunkyo-Ku, Tokyo, 113-8655 Japan; 3 Center for Disease Biology and Integrative Medicine, Graduate School of Medicine, The University of Tokyo, 7-3-1 Hongo, Bunkyo-Ku, Tokyo, 113-8655 Japan; 4 Department of Life Sciences, Graduate School of Arts and Sciences, The University of Tokyo, 3-8-1 Komaba, Meguro-Ku, Tokyo, 153-8902 Japan; 5 Japan Drug Development and Medical Affairs, Eli Lilly Japan K.K, 5-1-28 Isogami-Dori, Chuo-Ku, Kobe, 651-0086 Japan

**Keywords:** Rheumatoid arthritis, Pain-related behaviour, CAIA model, Baricitinib, JAK/STAT3 pathway

## Abstract

**Background:**

Janus kinase (JAK) inhibitors, such as baricitinib, are widely used to treat rheumatoid arthritis (RA). Clinical studies show that baricitinib is more effective at reducing pain than other similar drugs. Here, we aimed to elucidate the molecular mechanisms underlying the pain relief conferred by baricitinib, using a mouse model of arthritis.

**Methods:**

We treated collagen antibody-induced arthritis (CAIA) model mice with baricitinib, celecoxib, or vehicle, and evaluated the severity of arthritis, histological findings of the spinal cord, and pain-related behaviours. We also conducted RNA sequencing (RNA-seq) to identify alterations in gene expression in the dorsal root ganglion (DRG) following baricitinib treatment. Finally, we conducted in vitro experiments to investigate the direct effects of baricitinib on neuronal cells.

**Results:**

Both baricitinib and celecoxib significantly decreased CAIA and improved arthritis-dependent grip-strength deficit, while only baricitinib notably suppressed residual tactile allodynia as determined by the von Frey test. CAIA induction of inflammatory cytokines in ankle synovium, including interleukin (IL)-1β and IL-6, was suppressed by treatment with either baricitinib or celecoxib. In contrast, RNA-seq analysis of the DRG revealed that baricitinib, but not celecoxib, restored gene expression alterations induced by CAIA to the control condition. Among many pathways changed by CAIA and baricitinib treatment, the interferon-alpha/gamma, JAK-signal transducer and activator of transcription 3 (STAT3), and nuclear factor kappa B (NF-κB) pathways were considerably decreased in the baricitinib group compared with the celecoxib group. Notably, only baricitinib decreased the expression of colony-stimulating factor 1 (CSF-1), a potent cytokine that causes neuropathic pain through activation of the microglia–astrocyte axis in the spinal cord. Accordingly, baricitinib prevented increases in microglia and astrocytes caused by CAIA. Baricitinib also suppressed JAK/STAT3 pathway activity and *Csf1* expression in cultured neuronal cells.

**Conclusions:**

Our findings demonstrate the effects baricitinib has on the DRG in relation to ameliorating both inflammatory and neuropathic pain.

**Supplementary Information:**

The online version contains supplementary material available at 10.1186/s13075-024-03354-1.

## Background

Rheumatoid arthritis (RA) is a common disorder that occurs together with synovitis and subsequent joint destruction. Previous studies have revealed the involvement of inflammatory cytokines in this pathology, including interleukin (IL)-1β, IL-6, tumour necrosis factor (TNF) and potent signalling pathways, such as the nuclear factor kappa B (NF-κB) and Janus kinase (JAK)-signal transducer and activator of transcription 3 (STAT3) pathways. NF-κB is closely associated with both acute and chronic inflammatory responses. IL-1β and TNF are representative cytokines that activate NF-κB, which in turn upregulates IL-1β, TNF and IL-6 [1, 2]. IL-6 activates the JAK/STAT3 pathway and exerts various effects. IL-6 is also a downstream molecule of the JAK/STAT3 pathway, and the feedback loops of these cytokines and pathways enhance inflammation in RA [1, 2]. The JAK/STAT3 and NF-κB signalling pathways interact and work collaboratively with each other [3].

The management of RA has improved dramatically with the development of antibody-based drugs against TNF and IL-6 (known as biological disease-modifying antirheumatic drugs, DMARDs) and JAK inhibitors, in addition to methotrexate. However, residual pain continues to be observed in a large proportion of patients who are experiencing biological remission of RA [4]. It remains difficult to clearly assess and classify pain, although recent findings suggest the presence of neuropathic pain in rheumatic conditions [4].

Neuropathic pain arises from a complex process. The primary mediators derived from various cells sensitise sensory nerve endings and enhance the generation of secondary mediators from primary afferent neurons [5]. These secondary mediators, including colony-stimulating factor 1 (CSF-1), chemokine C–C motif ligand (CCL-21) and Wnt ligands, alter the properties of cells in the spinal dorsal horn (SDH) [5]. Microglia and astrocytes in the SDH are involved in the pathophysiology of neuropathic pain [6–9]. Generally, microglia respond rapidly to stimuli, becoming activated and proliferating, with astrocytes responding following microglia [10, 11]. The state during which astrocytes have proliferated and become activated is referred to as astrogliosis. Astrogliosis can persist even after the microglial response has subsided and is closely associated with neuropathic pain [11].

A recent clinical study indicated that baricitinib, an orally taken active, reversible selective inhibitor of JAK1 and JAK2, provides greater and more rapid pain relief than adalimumab, in addition to greater clinical improvement in patients with RA who had previously shown an inadequate response to methotrexate [12, 13]. The JAK/STAT3 pathway is required for nerve injury-induced astrocyte proliferation in the SDH of spinal nerve injury model rats [9]. Considering these findings, baricitinib may regulate pain in patients with RA independently of arthritis improvement by modulating the JAK/STAT3 pathway. However, the pathophysiological mechanism by which baricitinib relieves pain is not well understood.

Here, we investigate the molecular mechanisms underlying baricitinib-induced pain relief. We treated collagen antibody-induced arthritis (CAIA) model mice with baricitinib or celecoxib, a representative nonsteroidal anti-inflammatory drug that is widely used for the management of joint pain, and compared pain behaviours. We conducted RNA sequencing (RNA-seq) of the dorsal root ganglion (DRG) neurons to identify baricitinib-specific changes and the neuropathic pain mediators involved. We also undertook histological examinations of the SDH and directly observed the effects of baricitinib on cultured nerve cells.

## Methods

### Animals

All animal experiments were carried out in accordance with the Institutional Animal Care and Use Committee (IACUC) guidelines of The University of Tokyo. We complied with all relevant ethical regulations. Male DBA/1JJmsSlc mice aged 8 to 10 weeks were purchased from the Sankyo Labo Service Corporation (Tokyo, Japan).

### CAIA induction and drug administration

CAIA was induced by an intravenous injection of 1.5 mg anti-collagen monoclonal antibody cocktail (#53,040, Chondrex, WA, USA) at day 0 and intraperitoneal injection of 50 μg lipopolysaccharide from *E*. *coli* (#53,040, Chondrex) at day 3. The mice were divided into the following groups, which were compared with six mice per group: CAIA group (CAIA mice with administration of vehicle), baricitinib group (CAIA mice with administration of baricitinib), celecoxib group (CAIA mice with administration of celecoxib) and control group (wild-type mice with administration of vehicle). Baricitinib (4 mg/mL; HY-15315, Med Chem Express, NJ, USA) and celecoxib (3 mg/mL; C-2816, Tokyo Chemical Industry, Tokyo, Japan) were dissolved in distilled water, and 200 μL baricitinib (40 mg/kg/day) and celecoxib (30 mg/kg/day) were intragastrically administered once a day from day 0. The equivalent quantity of vehicle was administered to both the CAIA and control groups in the same manner. All tissue samples were harvested 2 h following the administration of the drugs or vehicle.

### Evaluation of arthritis

The severity of arthritis in each mouse’s paws was evaluated by visual scoring (arthritis score) and measurement of the joint width (paw width), according to a previously described scoring system [14]. The arthritis score is given as the total of all paw visual scores. The visual score for each paw, including digits, was graded on a scale of 0 to 4: 0, normal; 1, mild redness, slight swelling of the ankle or wrist; 2, moderate swelling of the ankle or wrist; 3, severe swelling, including some digits, ankles or feet; 4, maximally inflamed [14]. Thus, the maximum possible score per mouse was 16 [14]. Mice with an arthritis score of less than 4 at day 8 were excluded from all experiments. The paw width is given as the sum of the thickness of all wrists and the width of all ankles, measured using a digital calliper (C110T, Kloeplin, Schlüchtern, Germany) [15]. Measurements were taken every 2 to 3 days.

### Histological analysis

Two different sections of the L4-5 segments of the spinal cords and the ankles from each mouse were analysed. According to a previous study of vertebral landmarks for the identification of spinal cord segments in mice [16], L4 and L5 segments of the spinal cord were determined at the levels of the T12 DRG and the T13/L1 facet joints, respectively. All samples were fixed in 10% formalin at room temperature overnight, decalcified in 10% EDTA (pH 7.4) at 4℃ for 5 days, embedded in paraffin and cut into 5- or 6-μm sections. Haematoxylin and eosin (H&E) and safranin O staining were performed according to standard protocols. The degree of arthritis was histologically evaluated based on four categories: synovial inflammation (0–3), bone erosion (0–3), proteoglycan loss (0–3) and cartilage erosion (0–3), according to a previously described scoring system [17]. Synovial inflammation and bone erosion were evaluated by H&E staining, and proteoglycan loss and cartilage erosion were evaluated by safranin O staining [17]. Each category was graded from 0 (healthy) to 3 (severe), with incremental grading scores of 0.25 [17]. The total histological score was defined as the sum of the four category scores [17].

### Grip strength

Grip strength was measured using a digital grip-strength meter for mice (GPM-101B, Melquest, Toyama, Japan). Each mouse was gently placed on a wire mesh connected to a force transducer. After allowing the mouse to rest for 3 min, its tail was pulled and we continuously measured the force until the mouse released the wire mesh. This was repeated five times for each mouse, with intervals of more than 30 s. The grip strength power reported is the average of the maximum forces recorded.

### von Frey test

We evaluated tactile hypersensitivity using a dynamic plantar aesthesiometer (#37,550, Ugo Basile, Gemonio, Italy). Stimulation was performed as previously described [18]. A mouse was randomly placed in a box on a wire mesh, and the withdrawal pressure was measured at the mid-plantar area after a 30-min rest of behavioural accommodation. This measurement was repeated five times for both hind limbs of each mouse, with 2-min intervals, and the average values were reported.

### Quantitative reverse transcription-polymerase chain reaction (qRT-PCR)

Immediately after mice were euthanised and perfused with chilled phosphate buffered saline (PBS), bilateral ankles, L4-5 DRG and L4-5 spinal cord segments were harvested and placed in TRI Reagent (#TR118, Cosmo Bio, Tokyo, Japan). To obtain synovial tissue from the ankle joints, we removed the skin and tendon from the posterior aspect of the mouse ankle joint and then obtained soft tissue from behind the tibiotalar and talocalcaneal joints as the synovial tissue. Total RNA was purified using a Direct-zol RNA Kit (#R2062, Zymo Research, Irvine, CA, USA). The purified total RNA was reverse-transcribed into cDNA using ReverTra Ace qPCR RT Master Mix (#FSQ-201, TOYOBO, Osaka, Japan), and qRT-PCR was performed with THUNDERBIRD SYBR qPCR Mix (#FSQ-201, TOYOBO), using a Thermal Cycler Dice Real-Time System III (Takara Bio, Kusatsu, Japan). Relative quantification using a standard-curve method was used to compare gene expression levels. Target gene expression levels were normalised using glyceraldehyde-3-phosphate dehydrogenase (*Gapdh*) as an internal control. The primers used are listed in Supplementary Table 1.

### RNA sequencing

DRG samples were extracted from three mice per group on day 8. The purified total RNA was confirmed by the ratio of A260/A280 (1.8–2.0), and the RNA integrity number was > 7. Sequencing was performed on an Illumina NovaSeq 6000 System with two 150-bp paired-end reads and 26.7 million reads per sample were extracted. Quality checks and trimming were performed using fastp v0.23.4 [19]. Genome mapping was performed using STAR 2.7.10b [20]. The reference index was created using GRCm39.primaryassembly.genome.fa and gencode.vM31.primary_assembly.annotation.gtf, each downloaded from GENCODE [21]. Mapping information was quantified by RSEM v1.3.3 [22], then aggregated to count the matrix using script_rnakato [23]. Genes detected in 10 or more of the 12 samples were selected. The counts were normalised using DESeq2 [24]. Principal component analyses (PCAs) were performed using the prcomp in stats built in R 4.3.2 [25]. The variance (%) of the first and second principal components is shown in the PCA plot. Heatmaps were visualised using pheatmap: pretty heatmaps v1.0.12. In the four-group analysis, differentially expressed genes (DEGs) and their rankings were determined using TCC v1.42.0 [26] and EBSeq v2.0.0 [27]. The significant genes among CAIA + Baricitinib and others were extracted in Fig. 3G. The scores corresponding to each term in mSigDB (Gene Ontology (GO), m5.all.v2023.2.Mm.symbols.gmt; Pathway, m2.cp.v2023.2.Mm.symbols.gmt) were calculated sample-by-sample using AUCell v1.24.0. Differences among groups were determined using a one-way ANOVA, and significantly different GO terms and pathways were defined as *P*-values < 0.0001 and < 0.01, respectively. In the two-group analysis, DEGs were extracted using DESeq2 with the following cutoff values: adjusted *P*-values < 0.01 and log2Fc > 0.3. GO analyses were performed using clusterProfiler, referring to the same mSigDB as previously described. Bar plots of log10(p-value) values marked as positive or negative according to the sample were illustrated using ggplot2 [28]. Hallmark GO scores were calculated by referring to mh.all.v2023.2.Mm.symbols.gmt using AUCell. We then performed a t-test with a *P*-value < 0.05 considered to be significant. Gene set enrichment analysis (GSEA) of the Hallmark gene set was performed using GSEA 4.2.3 [29, 30].

### Immunohistochemistry

The paraffin-embedded spinal cord sections were incubated with primary antibodies against glial fibrillary acidic protein (GFAP, 1:2,000, 16,825–1-AP, Proteintech, Rosemont, IL, USA) or ionised calcium-binding adapter molecule 1 (Iba1, 1:250, NB100-1028, Novus Biologicals, Centennial, CO, USA) for 1 h at room temperature. The sections were then incubated for 30 min at room temperature with horseradish peroxidase-conjugated secondary antibodies, and Histofine Simple Stain DAB (3,3'-diaminobenzine) (#415,172, Nichirei Biosciences, Tokyo, Japan) was used for visualisation. Areas of positive signals in the bilateral spinal dorsal horn were measured using ImageScope (Leica Microsystems, Wetzlar, Germany) and compared using four-group comparisons [31].

### Cell culture

Cells of the mouse cell line Neuro-2a were purchased from the Japanese Collection of Research Bioresources (JCRB) Cell Bank. Neuro-2a cells were cultured in Minimum Essential Medium (#M5650, Sigma-Aldrich, St. Louis, MO, USA) with 10% fetal bovine serum (#F7524, Sigma-Aldrich), 1% penicillin–streptomycin (#09367–34, Nacalai Tesque, Kyoto, Japan) and 1% L-glutamine (#073–05391, Fujifilm Wako Pure Chemical Corporation, Osaka, Japan). Neuro-2a cells were seeded into 24-well plates at a density of 2.5 × 10^4^ cells per well with 500 μL of medium and incubated at 37 °C for 24 h. The Neuro-2a cells were then incubated with a mixture of recombinant human IL-6 (50 ng/mL, 206-IL, R&D Systems, Minneapolis, MN, USA) and recombinant human sIL-6R (50 ng/mL, 227-SR, R&D Systems) or recombinant human TNF-α (50 ng/mL, 300-01A, Peprotech, Rocky Hill, NJ, USA). Baricitinib (400 ng/mL, HY-15315, Med Chem Express), celecoxib (300 ng/mL; C-2816, Tokyo Chemical Industry) and BMS-345541 (50 ng/mL, S8044, Selleck Chemicals, Tx, USA) were added to the treatment groups.

### Statistical analysis

A one-way ANOVA followed by Tukey’s post hoc test was used to establish statistical significance in comparisons between the four groups, and values are expressed as means ± standard deviation (SD). A two-way ANOVA followed by Tukey’s post hoc test was used for time-course comparisons of the four groups, and the values are expressed as means ± standard error of the mean (SEM). The sample size was determined based on previous studies that employed similar methods. A *P*-value of less than 0.05 was considered to be statistically significant. The statistical analyses were performed using PRISM version 9.3.1 (GraphPad Software, San Diego, California, USA).

## Results

### Arthritis-induced neuropathic pain is suppressed by baricitinib

**Fig. 1:**
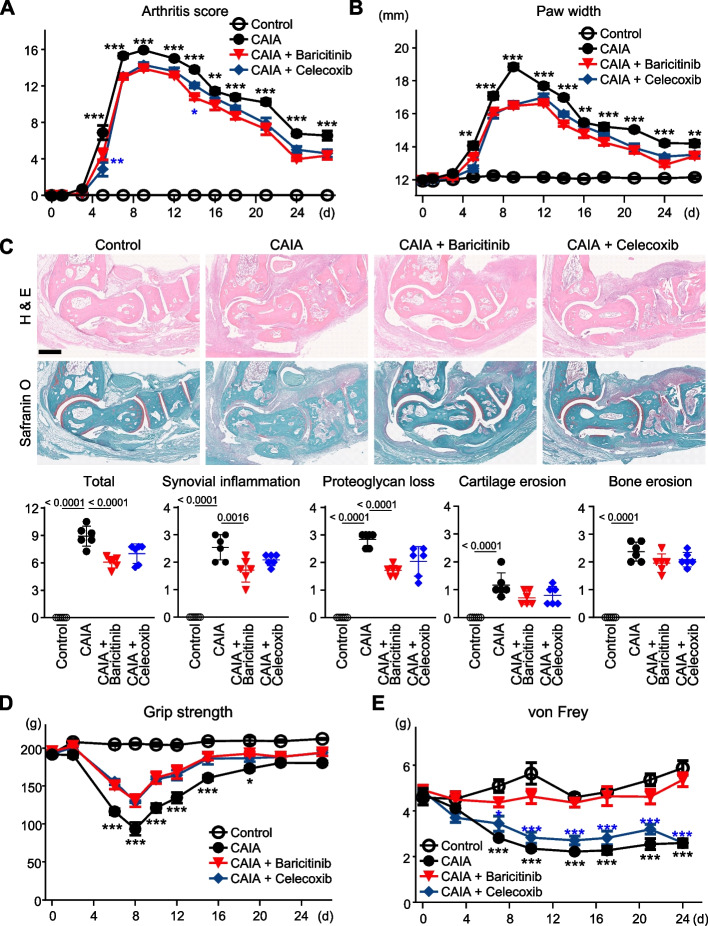
Arthritis transition of CAIA model mice. The transition of arthritis score (**A**) and paw width (**B**) of mice without CAIA induction (Control), CAIA-induced mice without treatment (CAIA), CAIA-induced mice treated with baricitinib (CAIA + Baricitinib) or celecoxib (CAIA + Celecoxib), during a four-week period following CAIA induction. (**C)** H&E and safranin O staining of ankle joints of the four groups at day 8. Scale bar, 5 mm. Lower panels indicate quantified scores for histological findings. (**D)** The transition of grip strength of the four groups over the four weeks. (**E)** The transition of withdrawal threshold of the four groups over the four weeks, based on von Frey test results. The data are expressed as line graphs and means ± SEM (A, B, D, E) or as dot plots and means ± SD (C) (*n* = 6 mice/group for A, B, D and E, and n = 6 sections with 3 mice/group for C). *P < 0.05, **P < 0.01, ***P < 0.005; two-way ANOVA (A, B, D, E), or *P*-values determined by one-way ANOVA (C) followed by Tukey’s post hoc test. Black and blue asterisks indicate the *P*-values of CAIA + Baricitinib vs CAIA, or CAIA + Baricitinib vs CAIA + Celecoxib, respectively

We initially examined pain-related behaviours in CAIA model mice treated with or without baricitinib or celecoxib. Arthritis, as determined by the gross appearance of the paw and the paw width, developed several days after being induced, reached a peak between days 7 and 10 and then gradually decreased (Fig. 1A and B). These arthritic changes were suppressed to a comparable extent by baricitinib or celecoxib treatment throughout almost the entire course of the treatment (Fig. 1A and B). Histological analysis of samples from day 8 indicated that both drugs suppressed synovial inflammation, including hyperplasia and cell infiltration, to a similar extent (Fig. 1C). Synovial inflammation and proteoglycan loss were improved by baricitinib, but the erosion of bone and cartilage was not significantly changed (Fig. 1C).

We next evaluated the pain-related behaviours in these groups. Grip strength is well known to correlate with pain in patients with RA and reflects real-time changes in joint inflammation in RA model mice [32]. The von Frey test is a representative method used to examine tactile allodynia, which is a cardinal feature of neuropathic pain [33]. In RA model mice, tactile allodynia evaluated by the von Frey test lasts after improvements in arthritis and grip-strength deficit [32]. Thus, we employed grip-strength and von Frey tests to evaluate any decrease in the arthritis-dependent pain threshold and residual tactile allodynia, respectively. Grip strength decreased until 8 days and began to increase thereafter in the CAIA group (Fig. 1D). The temporal evolution of this change exhibited a consistent concordance with the arthritis score and paw width (Fig. 1A-B and D). The decrease in grip strength caused by CAIA was significantly suppressed by treatment with baricitinib or celecoxib (Fig. 1D), exhibiting a consistent effect in line with their impact on arthritis (Fig. 1A and B). The nociceptive threshold as determined by the von Frey test showed a gradual decrease in the CAIA group (Fig. 1E). Notably, the changes in the threshold were remarkably suppressed by baricitinib treatment throughout the course of the treatment but were not suppressed by celecoxib (Fig. 1E). These data indicate that only baricitinib notably suppressed the tactile allodynia caused by arthritis, while arthritis-dependent pain was suppressed by both baricitinib and celecoxib.

### Inflammatory responses in ankle synovium are suppressed by baricitinib or celecoxib

**Fig. 2:**
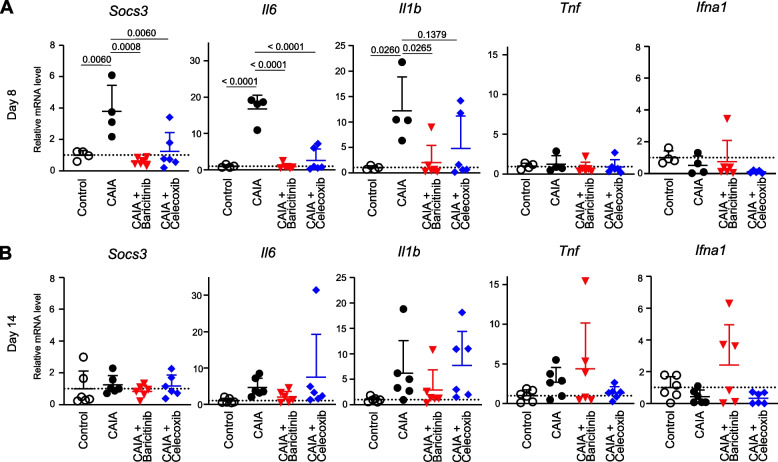
mRNA expression of marker genes in ankle synovium obtained from mice in the four groups: mice without CAIA induction (Control), CAIA-induced mice without treatment (CAIA), CAIA-induced mice treated with baricitinib (CAIA + Baricitinib) or celecoxib (CAIA + Celecoxib) at days 8 (**A**) and 14 (**B**). All data are expressed as dot plots and means ± SD. *P*-values were determined by one-way ANOVA followed by Tukey’s post hoc test

To investigate the effects of baricitinib and celecoxib at the molecular level, we quantified the mRNA expression of representative cytokine genes related to RA pathogenesis within the synovial tissue of ankle joints. At day 8, suppressor of cytokine signalling 3 (*Socs3*), which is a downstream molecule of the JAK/STAT3 pathway [34], *Il6* and *Il1b* were significantly induced by CAIA (Fig. 2A). The mRNA expression levels of these genes were suppressed by treatment with either baricitinib or celecoxib (Fig. 2A). The enhancements of these genes induced by CAIA had already subsided by day 14 (Fig. 2B). The expression of *Tnf* and interferon-alpha (*Ifna1*) was not significantly increased in response to CAIA at days 8 or 14 (Fig. 2A and B). Interferon-gamma (*Ifng*) gene expression was barely detectable.

### Comprehensive analysis of mRNA in DRG

**Fig. 3:**
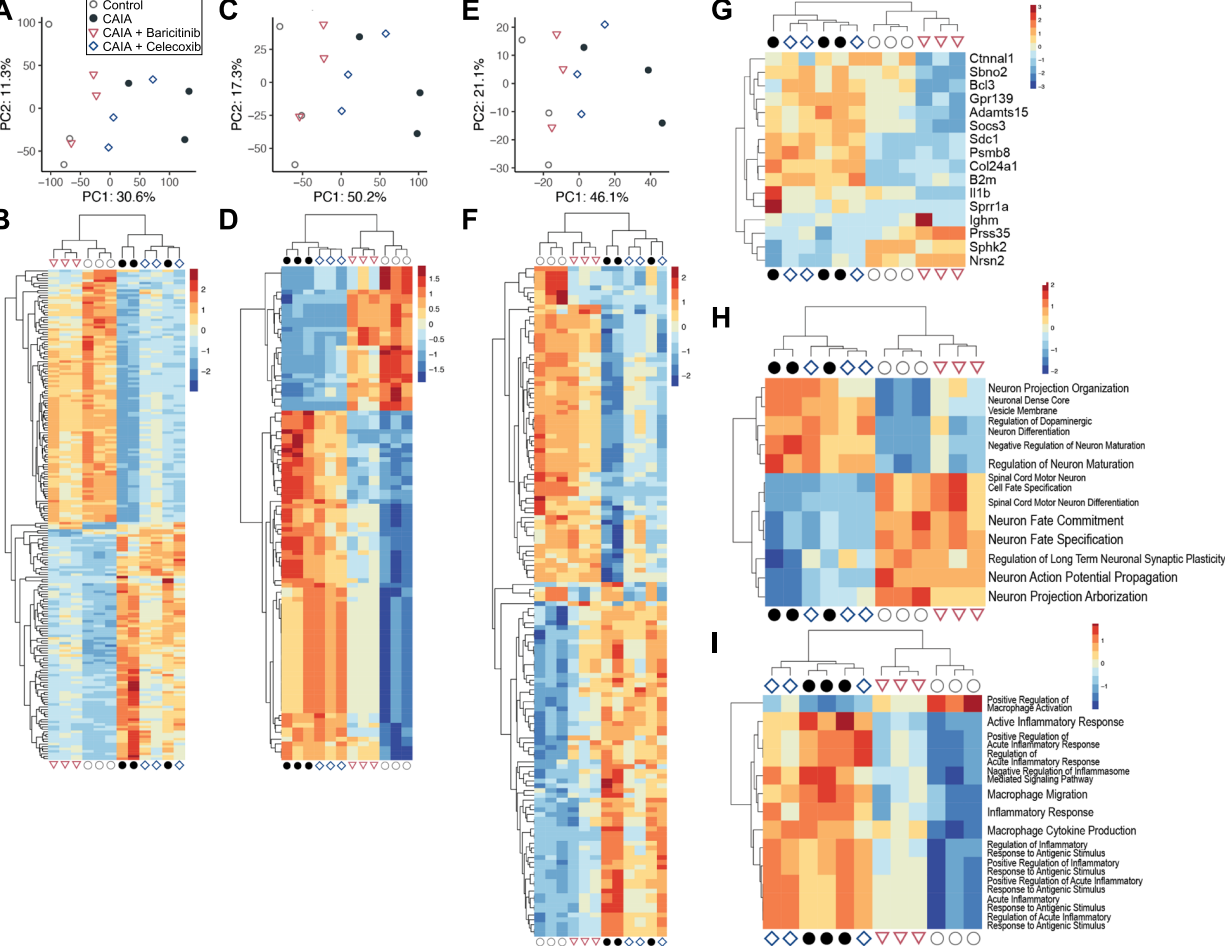
Comprehensive RNA-seq analyses of gene expression in the DRG. PCA (**A, C, E**) and heatmaps (**B, D, F**) of DEGs in DRG samples of the four groups at day 8 (*n* = 3 mice/group). The analyses were based on genes (**A** and **B**), GO terms (**C** and **D**) and pathways (**E** and **F**). All significantly changed genes (**B**), GO terms (**D**) and pathways (**F**) are shown in the heatmaps. Heatmaps with the names of genes, GO terms and pathways can be found in Supplementary Fig. 1–3. (**G)** A heatmap of genes that were significantly altered in the CAIA + Baricitinib group. (**H)** Heatmaps of neuron-related GO terms that were significantly altered in the CAIA + Baricitinib group. (**I)** Heatmaps of macrophage inflammation-related GO terms that were significantly altered in the CAIA + Baricitinib group. The colours of the heatmaps represent the expression levels of each gene or gene set. High levels of expression are depicted in red, while low levels of expression are depicted in blue

For a comprehensive understanding of the effects of baricitinib or celecoxib on gene expression in DRG cells, we next performed RNA-seq analysis of DRG obtained from mice at day 8. All PCAs and heatmap analyses based on genes, GO terms and pathways revealed that the gene expression patterns of the baricitinib and celecoxib groups were similar to those of the control and CAIA groups, respectively (Fig. 3A-F, Supplementary Fig. 1–3). Notably, all heatmaps clearly indicated that the four groups could be divided into two groups: the control and baricitinib groups vs the CAIA and celecoxib groups (Fig. 3B, D, F Supplementary Fig. 1–3). We further listed upregulated genes, GO terms and pathways that were significantly altered in the baricitinib group (Fig. 3G-I). *Socs3* and *Il1b* were detected as genes significantly altered by baricitinib treatment (Fig. 3G). In particular, terms related to neurons and macrophage inflammation were altered in the baricitinib group, with a similar trend observed in the control group (Fig. 3H and I).

**Fig. 4:**
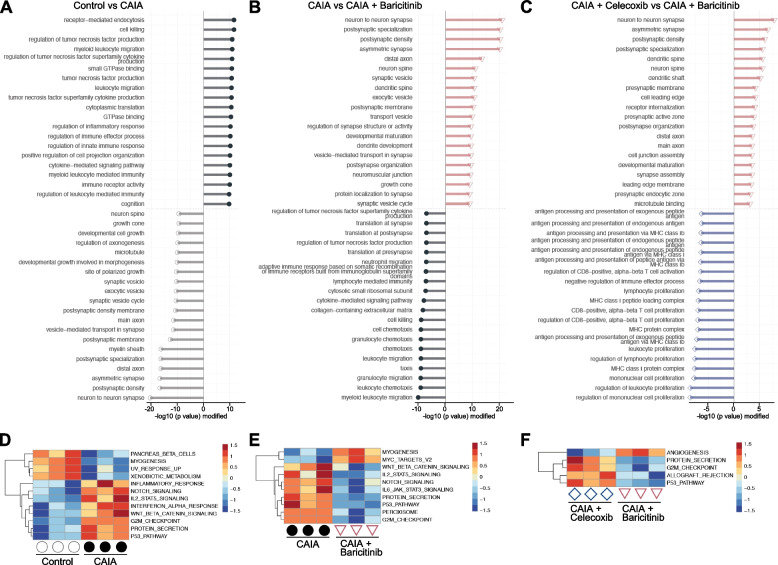
Enriched terms and pathways between respective two groups. Distinct top-20 GO terms in DRGs between Control vs CAIA (**A**), CAIA vs CAIA + Baricitinib (**B**), and CAIA + Celecoxib vs CAIA + Baricitinib (**C**), determined using by clusterProfiler. Heatmaps of significantly enriched Hallmark pathways in DRG between Control vs CAIA (**D**), CAIA vs CAIA + Baricitinib (**E**), and CAIA + Celecoxib vs CAIA + Baricitinib (**F**)

We next compared terms and pathways between two groups: the control vs CAIA groups, the CAIA vs baricitinib groups, and the celecoxib and baricitinib groups, based on the DEGs identified through the four-group comparisons. According to a clusterProfiler analysis comparing the control and CAIA groups, inflammation- and immune-related terms were enriched in the CAIA group, while those related to physiological development or maintenance of neuronal systems were enriched in the control group (Fig. 4A). Some of these neuron-related terms, and immune-related terms, including chemotaxis, were elevated in the baricitinib and CAIA groups, respectively (Fig. 4B). Of note and similar to the comparison between the CAIA and baricitinib groups, neuron-related terms were enriched in the baricitinib group, while immune-related terms were enriched in the celecoxib group (Fig. 4C). Hallmark pathway analyses [35] indicated the upregulation of various pathways by CAIA, such as inflammatory response, Notch, Wnt, interferon-alpha and p53, while many of them were downregulated by baricitinib (Fig. 4D and E). The IL-6/JAK/STAT3 signalling pathway was one of the pathways downregulated by baricitinib, but Hallmark pathway analysis showed that these changes were not significantly different between the baricitinib and celecoxib groups (Fig. 4E and F).

**Fig. 5:**
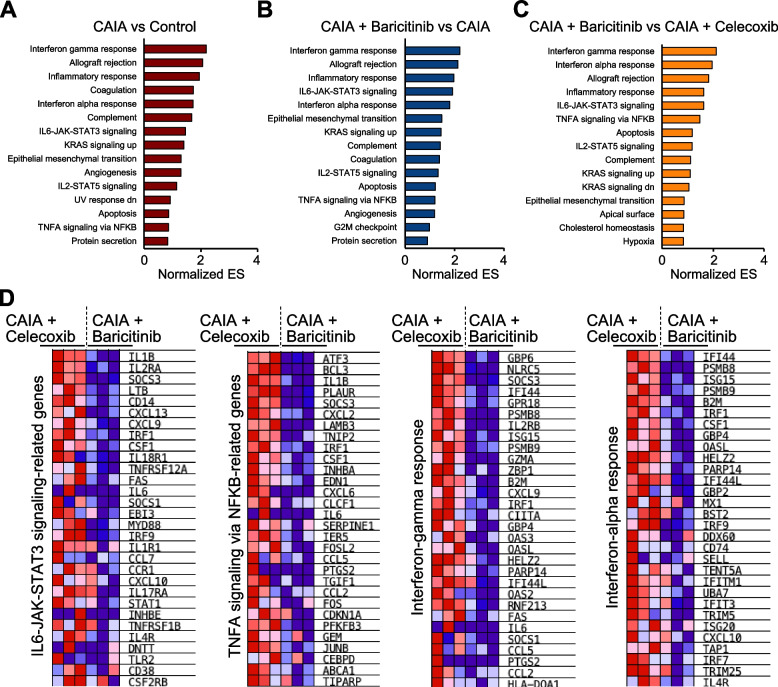
Pathway analyses by gene set enrichment analysis (GSEA). Altered pathways in DRG between Control vs CAIA (**A**), CAIA + Baricitinib vs CAIA (**B**), and CAIA + Baricitinib vs CAIA + Celecoxib (**C**). (**D)** Heatmaps of the top-30 ranked genes in each Hallmark pathway related to the JAK/STAT3, NK-κB, and interferon-gamma or -alpha pathways

To verify pathway alterations in the DRG, we also conducted GSEA, another method commonly used for pathway analyses. Various pathways were changed in the three sets of comparisons between two groups (Fig. 5A-C). Among them, the interferon-gamma/alpha response, the IL-6/JAK/STAT3 signalling, and the TNFA-signalling via NF-κB pathways were more enriched in the baricitinib group than in the celecoxib group (Fig. 5C). The top-30 ranked genes in each pathway are presented as heatmaps (Fig. 5D). In addition to pathogenesis-related cytokines such as *Il1b* and *Il6*, activating transcription factor 3 (*Atf3*), a rapidly expressed marker of nerve injury, and *Socs3* were decreased by baricitinib (Fig. 5D). Interestingly, *Csf1*, a representative secondary mediator of neuropathic pain, was also downregulated in the baricitinib group (Fig. 5D). The RNA-seq analyses suggested that baricitinib treatment changed the gene expression profiles of DRG in the CAIA mice more closely to the control group than the celecoxib treatment via modulating several signalling pathways associated with RA, including IL-6/JAK/STAT3.

### JAK/STAT3 pathway and microglia and astrocyte stimulators are specifically suppressed by baricitinib in DRG

**Fig. 6:**
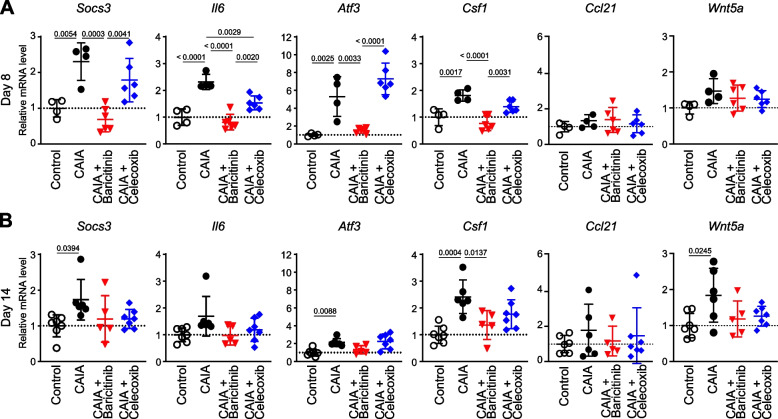
mRNA levels of marker genes in the DRG. qRT-PCR data of DRG from the four groups at days 8 (*n* = 4–6 mice/group) (**A**) and 14 (*n* = 5–7 mice/group) (**B**). All data are expressed as dot plots and means ± SD. *P*-values were determined by one-way ANOVA followed by Tukey’s post hoc test

Next, we used qRT-PCR to investigate mRNA expression in DRG. At day 8, the CAIA-induced expression of *Socs3* and *Il6* was more efficiently suppressed by baricitinib than by celecoxib (Fig. 6A). This was in agreement with the aforementioned RNA-seq data and an earlier finding that the IL-6/JAK/STAT3 pathway enhances IL-6 expression [36]. The expression of *Atf3* was also enhanced by CAIA induction and efficiently suppressed only by baricitinib (Fig. 6A). The mRNA levels of these marker genes were not significantly different between the CAIA and celecoxib groups, except for *Il6* (Fig. 6A). By day 14, the changes in these markers had subsided (Fig. 6B).

We further investigated whether primary afferent neuron-derived neuropathic pain mediators were changed by treatment with baricitinib. According to the RNA-seq data, the CAIA-induced expression of *Csf1* was downregulated more by baricitinib than it was by celecoxib (Fig. 6A and B). The expression of other activators for microglia, such as *Ccl21* and *Wnt5a,* was not obviously changed by CAIA or baricitinib (Fig. 6A and B). *Wnt3a* expression was barely detectable in DRG.

### Proliferation of microglia and astrocytes in the SDH is suppressed by baricitinib

**Fig. 7:**
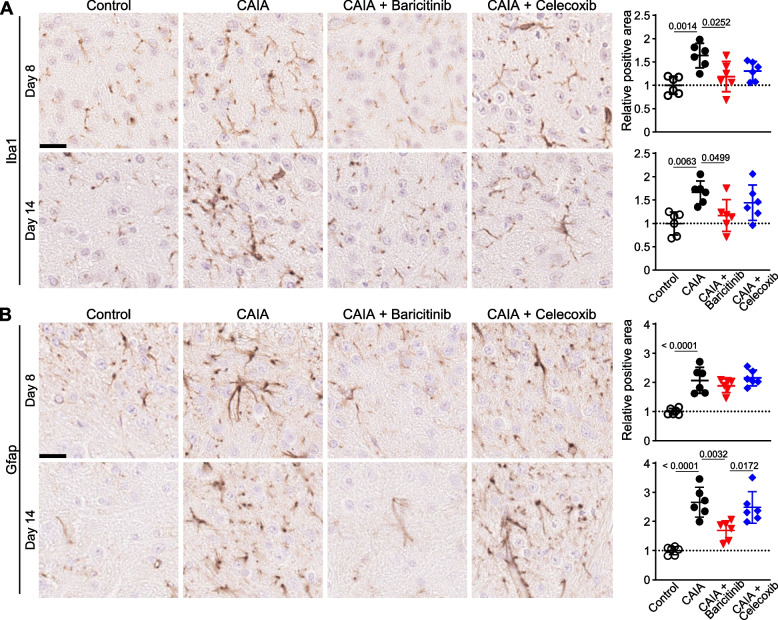
Alteration of microglia and astrocytes in the SDH. Immunohistochemistry for the microglia marker Iba1 (**A**) and the astrocyte marker Gfap (**B**) in the SDH of the four groups at days 8 and 14. Scale bar, 20 µm. The right-hand panels indicate positive areas relative to the control (*n* = 6 sections with 3 mice/group). All data are expressed as dot plots and means ± SD. *P*-values were determined by one-way ANOVA followed by Tukey’s post hoc test

Next, we histologically analysed the SDH. Iba1-positive microglia were increased by CAIA at both days 8 and 14 (Fig. 7A). Only baricitinib treatment significantly inhibited the increase in microglial proliferation induced by CAIA at days 8 and 14 (Fig. 7A). GFAP-positive astrocytes were increased by CAIA and were not suppressed by baricitinib or celecoxib treatment at day 8 (Fig. 7B). However, at day 14, the number of astrocytes was efficiently decreased by baricitinib, while there was no significant difference between the CAIA and celecoxib groups (Fig. 7B). These histological findings suggested that neuropathic pain-related changes in the spinal cord were only suppressed by baricitinib, at least on day 14.

### Direct effects of baricitinib on nerve cells

**Fig. 8:**
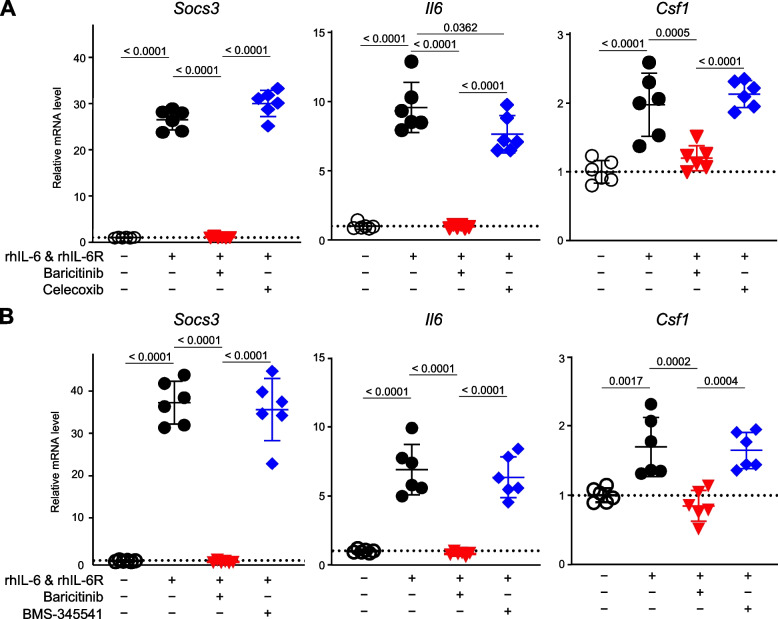
mRNA levels of *Socs3*, *Il6* and *Csf1* in Neuro-2a cells. Neuro-2a cells were cultured with or without 50 ng/mL rhIL-6, 50 ng/mL rhIL-6R, 400 ng/mL baricitinib, and 300 ng/mL celecoxib (**A**), or 50 ng/mL BMS (**B**) for 8 h (n = 6 biological replicates/group). All data are expressed as dot plots and means ± SD. *P*-values were determined by one-way ANOVA followed by Tukey’s post hoc test

Our in vivo data strongly suggest that baricitinib suppresses tactile allodynia primarily through the targeting of CSF-1 production in the DRG. To investigate whether baricitinib directly affects nerve cells in the DRG, we examined the effects of baricitinib on nerve cells in vitro. We treated cells of Neuro-2a, a commonly used neuronal cell-line derived from mouse neuroblastoma, with rhIL-6 and rhIL-6R, or rhTNF-α. When the Neuro-2a cells were treated with rhIL-6 and rhIL-6R, *Csf1* expression was upregulated, in accordance with the remarkable changes seen in *Socs3* and *Il6* expression (Fig. 8A). These changes in expression levels were significantly suppressed by baricitinib but not by celecoxib (Fig. 8A). When Neuro-2a cells were treated with rhTNF-α, the expression levels of *Il6* and *Csf1*, but not *Socs3*, were upregulated (Supplementary Fig. 4A). However, neither baricitinib nor celecoxib suppressed these changes (Supplementary Fig. 4A). When we added BMS-345541, a potent I-kappaB kinase inhibitor, to the Neuro-2a cells stimulated with rhTNFα, *Il6* and *Csf1* expression levels were clearly suppressed by the addition of more than 50 ng/mL BMS-345541 (Supplementary Fig. 4B), while the increased gene expression resulting from stimulation with rhIL-6 and rhIL-6R was not affected by 50 ng/mL BMS-345541 (Fig. 8B). These in vitro data confirmed that baricitinib suppresses the expression of *Il6* and *Csf1* induced by the IL-6/JAK/STAT3 pathway in nerve cells.

## Discussion

Here, we have shown that CAIA mice exhibit both arthritis-dependent pain and residual tactile allodynia. CAIA-induced grip-strength deficit was improved by treatment with either baricitinib or celecoxib, but tactile allodynia was notably suppressed only by baricitinib. As well as arthritis, inflammatory responses in ankle synovium were significantly suppressed by both baricitinib and celecoxib. RNA-seq analysis of DRG revealed that baricitinib, but not celecoxib, restored gene expression alterations induced by CAIA to the control condition. Among many pathways changed by CAIA and baricitinib treatment, the JAK/STAT3 and NF-κB pathways were decreased in the baricitinib group compared with the celecoxib group. Notably, only baricitinib decreased the expression of CSF-1 in the DRG. Accordingly, baricitinib prevented increases in microglia and astrocytes caused by CAIA. JAK/STAT3 pathway activity and *Csf1* expression were suppressed by baricitinib in cultured neuronal cells. These findings indicate that baricitinib ameliorates CAIA-induced pain by suppressing the IL-6/JAK/STAT3 pathway and further expression of CSF-1 in the DRG.

Despite major advances in the treatment of RA in recent years, dealing with residual pain remains a major challenge. Fautrel et al. used a matching-adjusted indirect comparison to compare the pain-reducing ability of baricitinib and other comparators and concluded that baricitinib monotherapy demonstrated greater pain reduction compared with tocilizumab and adalimumab monotherapy [37]. In the present study, the results of the grip strength test were closely associated with the degree of arthritis, while the decrease in pain threshold as determined by the von Frey test, an indicator of allodynia, was not improved even after the arthritis had subsided (Fig. 1A-E). The superior effects of baricitinib for pain relief in patients with RA are also supported by our current data linked to pain-related behaviours and our histological findings of the SDH (Figs. 1D, E, and 7). It should be noted that we did not compare baricitinib with biological DMARDs, including antibodies for TNF or IL-6, mainly because mice antibodies are not comparable with those used in humans. Despite this limitation, the superiority of baricitinib in the amelioration of pain threshold decrease in mouse arthritis models might be observed in comparison with the inhibitors of these cytokines [38], because their expression in the synovium was efficiently suppressed by celecoxib, as well as by baricitinib (Fig. 2A).

Tsuda et al. reported that the JAK/STAT3 pathway is activated in a rat spinal nerve injury model and plays essential roles in the development of astrogliosis and neuropathic pain [9]. Additionally, intrathecal administration of JAK2/3 inhibitors has been shown to improve astrocyte proliferation and alleviate pain behaviours [9]. However, the involvement of peripheral nerve cells or the JAK/STAT3 pathway in these cells was not evaluated [9]. We therefore sought to determine which tissue was the most important to enable baricitinib to suppress tactile allodynia. In general, most systemically administered chemical compounds can barely cross the blood–brain barrier (BBB). However, using a mouse RA model, Matsushita et al. showed that baricitinib acts on a part of brain where the BBB is weak [39]. In our present data, baricitinib treatment, more than celecoxib treatment, altered the pattern of gene expression in the DRG of CAIA mice in a way similar to that seen in the control group, although both treatments did improve arthritis. *Socs3* expression in DRG and Neuro-2a cells was noticeably decreased only by baricitinib and not by celecoxib (Fig. 6A and 8). Several limitations of our study should be noted. (1) We could not clearly distinguish inflammatory and neuropathic pain by our current technique of using pain-related behaviours. (2) We could not directly determine how baricitinib was delivered to the synovium, DRG or SDH. (3) We could not perform in vitro experiments using DRG-derived primary cells. Nevertheless, our data indicate that baricitinib exerts specific effects on nerve cells in the DRG and that these effects were responsible for the difference between the baricitinib and celecoxib groups.

In the current study, both the JAK/STAT3 and NF-κB pathways in the DRG were suppressed more efficiently by baricitinib than they were by celecoxib (Fig. 5C and D). *Il6* is a target gene of both the STAT3 and NF-κB pathways, and IL-6 induces further activation of the JAK/STAT3 pathway [36, 40]. Our RNA-seq data showed that, compared with celecoxib, baricitinib downregulated *Il1b* (Fig. 5D). Considering that IL-1β is both an upstream and downstream gene of NF-κB [40], the present data indicate that baricitinib suppressed the IL-6 Amp [41], the amplification of these two pathways mediated by IL-6, during arthritis. On the other hand, we could not precisely determine the cell types involved in the IL-6 Amp. Previous studies have shown IL-6 expression in the DRG, with some demonstrating its expression in neurons [42–44]. However, the DRG includes various types of immune cells and neuronal cells other than neurons. It remains unclear which types of cells are biologically effective as providers of IL-6 in these processes. It should also be noted that the IL6-JAK-STAT3 pathway was not significantly detected by Hallmark pathway analysis (Fig. 4F), but it was significantly detected by GSEA (Fig. 5C). We hypothesise two reasons for this inconsistency: (1) differences in the algorithm between Hallmark pathway analysis and GSEA, and (2) differences in the number of groups analysed. For (1), the Hallmark pathway analysis was based on the quantity of gene expression levels, while the GSEA was based on the ranking of gene expression levels. For (2), the Hallmark pathway analysis was conducted based on four-group data analyses, while GSEA was conducted based on two-group data analyses. Such differences may have contributed to the issue mentioned above.

When proteins secreted in response to inflammation sensitise sensory nerve endings, primary afferent neurons begin to increase the production of secondary mediators, including CSF-1, CCL-21, WNT-3A and WNT-5A, which contributes to the activation of microglia in the SDH and the development of neuropathic pain [5]. Among these secondary mediators, only *Csf1* is upregulated in the DRG by CAIA and selectively suppressed by baricitinib (Fig. 6A and B). CSF-1 is the best characterised among the primary afferent neuron-derived secondary mediators for microglial activation [5]. CSF-1 is induced by inflammatory mediators, such as IL-1β, from satellite cells and macrophages around the DRG [5]. Yu et al. showed that *Csf1* depletion in sensory neurons largely abrogated nerve injury-induced microglial activation and proliferation [45]. *Csf1* is one of the transcriptional target genes of NF-κB [40, 46]. Additionally, *Csf1* is induced by STAT3, while CSF-1, as well as IL-6, can activate the STAT3 pathway [47, 48]. Taken together, these data suggest that baricitinib greatly suppressed arthritis-induced inflammatory and neuropathic pain through the inhibition of IL-6 Amp and the subsequent decrease in *Csf1* expression in DRG.

## Conclusions

We found that baricitinib suppresses prolonged spinal glial cell activity and persistent pain threshold decrease in CAIA mice; furthermore, it inhibited the upregulation of IL-6 Amp and CSF-1 in DRG. These findings may contribute to improved management of residual pain in patients with RA and the further development of novel methods for pain management.

### Supplementary Information

Additional file 1. Supplementary Figure 1. Heatmap of the top-200 significantly altered genes among the four groups. The heatmap without gene names is shown in Fig. 3B

Additional file 2. Supplementary Figure 2. Heatmap of the top-106 significantly altered GO biological processes terms among the four groups. The heatmap without term names is shown in Fig. 3D

Additional file 3. Supplementary Figure 3. Heatmap of the top-140 significantly altered pathways among the four groups. The heatmap without pathway names is shown in Fig. 3F

Additional file 4. Supplementary Figure 4. mRNA levels of Socs3, Il6 and Csf1 in Neuro-2a cells. Neuro-2a cells were cultured with or without 50 ng/mL rhTNF (**A**, **B**), 400 ng/mL baricitinib (**A**), 300 ng/mL celecoxib (A) or 50 ng/mL BMS (**B**) for 8 hours. All data are expressed as dot plots and means ± SD. P-values were determined by one-way ANOVA followed by Tukey’s post hoc test

Additional file 5.

## Data Availability

The data underlying this article are available in the DNA Data Bank of Japan Sequence Read Archive (DRA) at https://www.ddbj.nig.ac.jp/dra/index.html, and can be accessed with accession number PRJDB16633.

## Abbreviations

ATF: Activating transcription factor
BBB: Blood–brain barrier
CAIA: Collagen antibody-induced arthritis
CCL: Chemokine C–C motif ligand
CSF: Colony-stimulating factor
DEG: Differentially expressed gene
DMARD: Disease-modifying antirheumatic drug
DRA: DNA Data Bank of Japan Sequence Read Archive
DRG: Dorsal root ganglion
GAPDH: Glyceraldehyde-3-phosphate dehydrogenase
GSEA: Gene set enrichment analysis
GO: Gene Ontology
H&E: Haematoxylin and eosin
IACUC: Institutional Animal Care and Use Committee
IFNα: Interferon alpha
IFNγ: Interferon gamma
IL: Interleukin
JAK: Janus kinase
JCRB: Japanese Collection of Research Bioresources
NF-κB: Nuclear factor kappa B
PBS: Phosphate buffered saline
PCA: Principal component analysis
qRT-PCR: Quantitative reverse transcription-polymerase chain reaction
RA: Rheumatoid arthritis
RNA-seq: RNA sequencing
SD: Standard deviation
SDH: Spinal dorsal horn
SOCS: Suppressor of cytokine signalling
STAT: Signal transducer and activator of transcription
SEM: Standard error of the mean
TNF: Tumour necrosis factor
