# Supplementary material for: Baricitinib ameliorates inflammatory and neuropathic pain in collagen antibody-induced arthritis mice by modulating the IL-6/JAK/STAT3 pathway and CSF-1 expression in dorsal root ganglion neurons
Source: Arthritis Res Ther. 2024 Jun 15;26:121. doi: 10.1186/s13075-024-03354-1 (PMC11179219; doi:10.1186/s13075-024-03354-1)
Supplement: Supplementary file 1 — Additional file 1. Supplementary Figure 1. Heatmap of the top-200 significantly altered genes among the four groups. The heatmap without gene names is shown in Fig. 3B [file 13075_2024_3354_MOESM1_ESM.pptx]

## Slide 1
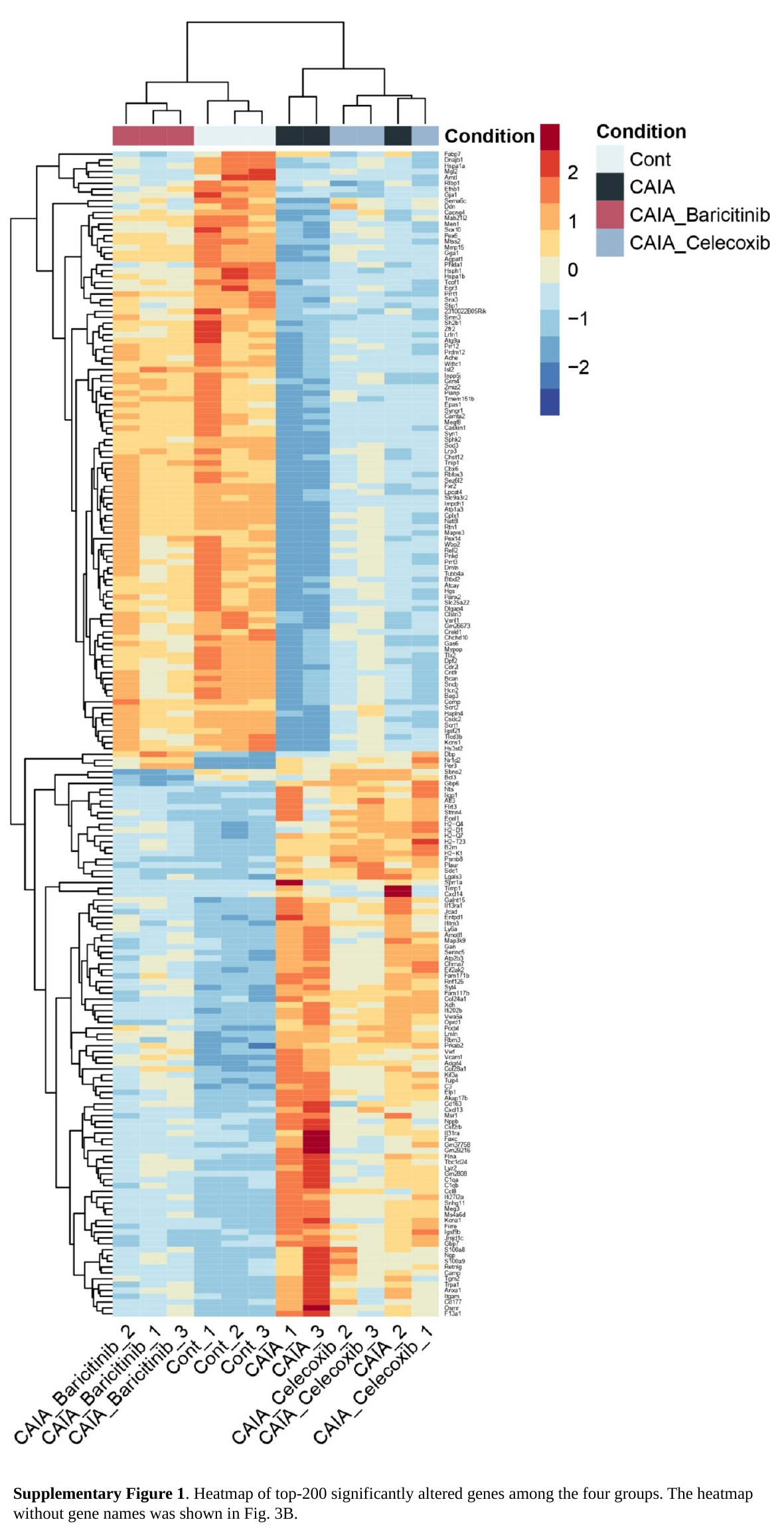

Supplementary Figure 1. Heatmap of top-200 significantly altered genes among the four groups. The heatmap without gene names was shown in Fig. 3B.
