# Supplementary material for: Baricitinib ameliorates inflammatory and neuropathic pain in collagen antibody-induced arthritis mice by modulating the IL-6/JAK/STAT3 pathway and CSF-1 expression in dorsal root ganglion neurons
Source: Arthritis Res Ther. 2024 Jun 15;26:121. doi: 10.1186/s13075-024-03354-1 (PMC11179219; doi:10.1186/s13075-024-03354-1)
Supplement: Supplementary file 2 — Additional file 2. Supplementary Figure 2. Heatmap of the top-106 significantly altered GO biological processes terms among the four groups. The heatmap without term names is shown in Fig. 3D [file 13075_2024_3354_MOESM2_ESM.pptx]

## Slide 1
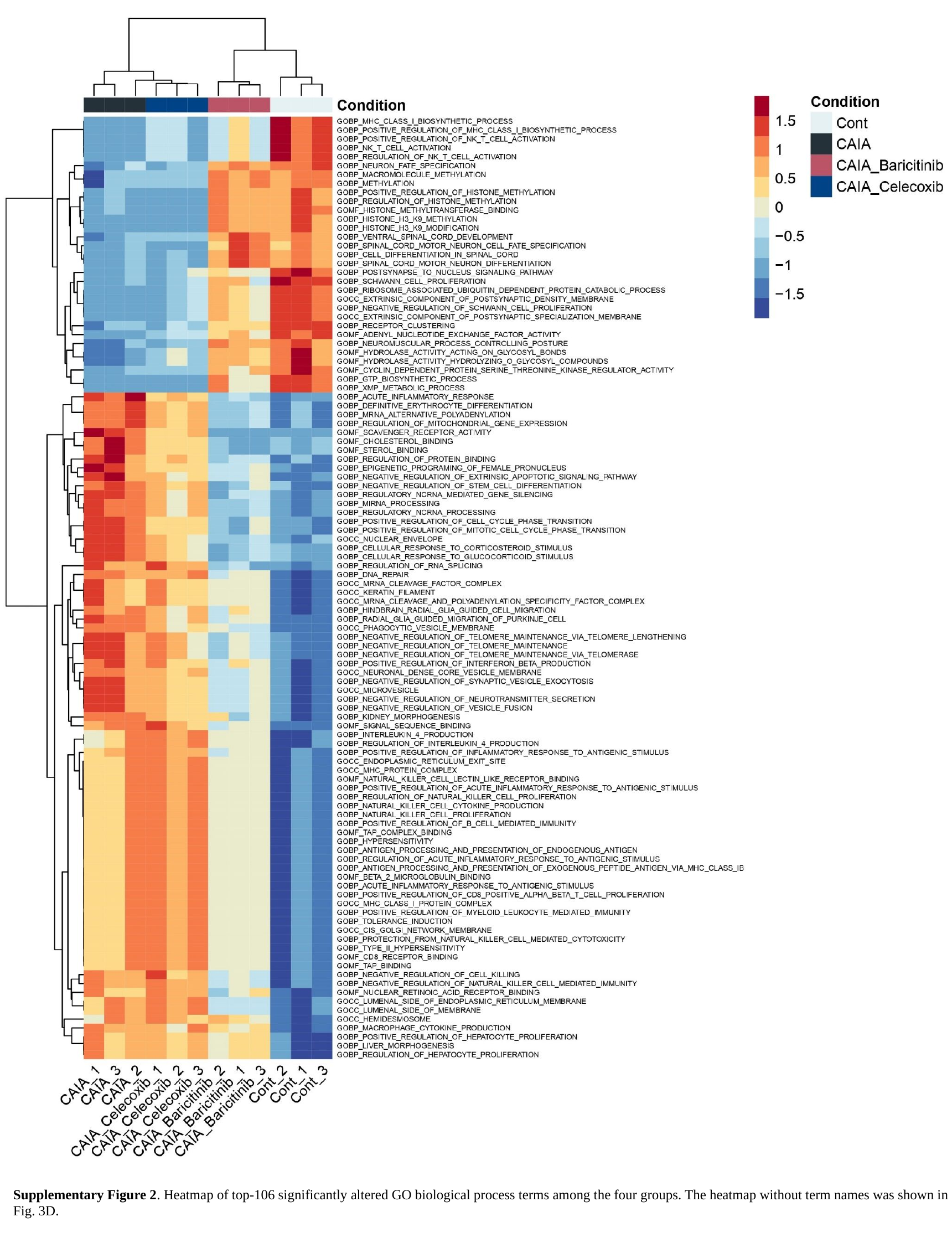

Supplementary Figure 2. Heatmap of top-106 significantly altered GO biological process terms among the four groups. The heatmap without term names was shown in Fig. 3D.
