# Supplementary material for: Baricitinib ameliorates inflammatory and neuropathic pain in collagen antibody-induced arthritis mice by modulating the IL-6/JAK/STAT3 pathway and CSF-1 expression in dorsal root ganglion neurons
Source: Arthritis Res Ther. 2024 Jun 15;26:121. doi: 10.1186/s13075-024-03354-1 (PMC11179219; doi:10.1186/s13075-024-03354-1)
Supplement: Supplementary file 4 — Additional file 4. Supplementary Figure 4. mRNA levels of Socs3, Il6 and Csf1 in Neuro-2a cells. Neuro-2a cells were cultured with or without 50 ng/mL rhTNF (A, B), 400 ng/mL baricitinib (A), 300 ng/mL celecoxib (A) or 50 ng/mL BMS (B) for 8 hours. All data are expressed as dot plots and means ± SD. P-values were determined by one-way ANOVA followed by Tukey’s post hoc test [file 13075_2024_3354_MOESM4_ESM.pptx]

## Slide 1
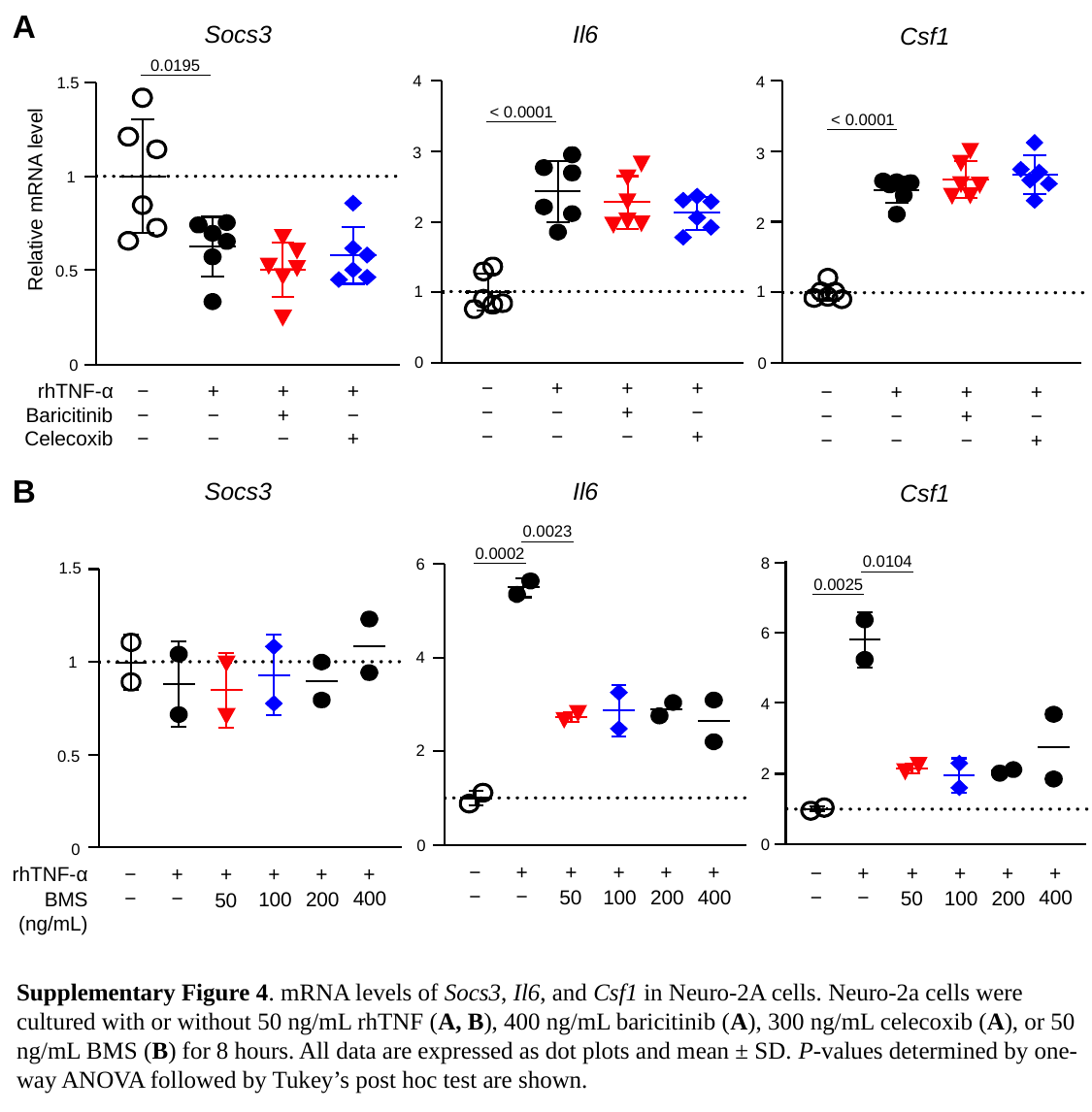

A
Socs3
Il6
Csf1
0.0195
4
4
1.5
< 0.0001
< 0.0001
3
3
1
Relative mRNA level
2
2
0.5
1
1
0
0
0
−
+
+
+
−
−
+
−
−
−
−
+
rhTNF-α
−
+
+
+
−
+
+
+
−
−
+
−
−
−
−
+
Baricitinib
−
−
+
−
Celecoxib
−
−
−
+
B
Socs3
Il6
Csf1
0.0023
0.0002
0.0104
8
6
1.5
0.0025
6
4
1
4
2
0.5
2
0
0
0
−
+
+
+
+
+
−
−
100
200
400
50
−
+
+
+
+
+
−
−
400
100
200
50
rhTNF-α
−
+
+
+
+
+
−
−
400
100
200
50
BMS
(ng/mL)
Supplementary Figure 4. mRNA levels of Socs3, Il6, and Csf1 in Neuro-2A cells. Neuro-2a cells were cultured with or without 50 ng/mL rhTNF (A, B), 400 ng/mL baricitinib (A), 300 ng/mL celecoxib (A), or 50 ng/mL BMS (B) for 8 hours. All data are expressed as dot plots and mean ± SD. P-values determined by one-way ANOVA followed by Tukey’s post hoc test are shown.
