# Supplementary material for: Baricitinib ameliorates inflammatory and neuropathic pain in collagen antibody-induced arthritis mice by modulating the IL-6/JAK/STAT3 pathway and CSF-1 expression in dorsal root ganglion neurons
Source: Arthritis Res Ther. 2024 Jun 15;26:121. doi: 10.1186/s13075-024-03354-1 (PMC11179219; doi:10.1186/s13075-024-03354-1)
Supplement: Supplementary file 5 — Additional file 5. [file 13075_2024_3354_MOESM5_ESM.pptx]

## Slide 1
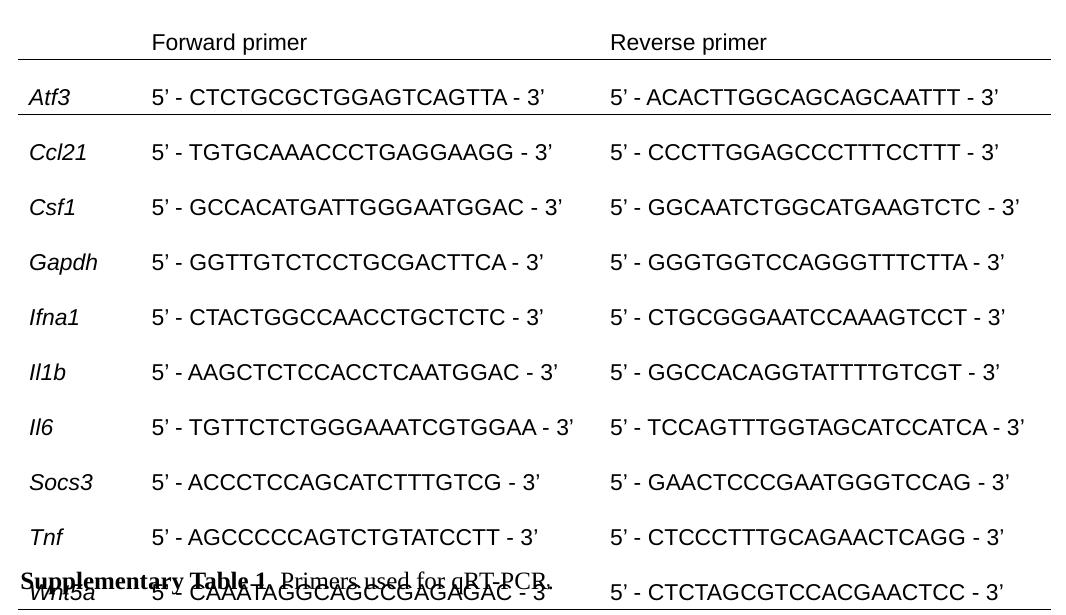

| | Forward primer | Reverse primer |
| --- | --- | --- |
| Atf3 | 5’ - CTCTGCGCTGGAGTCAGTTA - 3’ | 5’ - ACACTTGGCAGCAGCAATTT - 3’ |
| Ccl21 | 5’ - TGTGCAAACCCTGAGGAAGG - 3’ | 5’ - CCCTTGGAGCCCTTTCCTTT - 3’ |
| Csf1 | 5’ - GCCACATGATTGGGAATGGAC - 3’ | 5’ - GGCAATCTGGCATGAAGTCTC - 3’ |
| Gapdh | 5’ - GGTTGTCTCCTGCGACTTCA - 3’ | 5’ - GGGTGGTCCAGGGTTTCTTA - 3’ |
| Ifna1 | 5’ - CTACTGGCCAACCTGCTCTC - 3’ | 5’ - CTGCGGGAATCCAAAGTCCT - 3’ |
| Il1b | 5’ - AAGCTCTCCACCTCAATGGAC - 3’ | 5’ - GGCCACAGGTATTTTGTCGT - 3’ |
| Il6 | 5’ - TGTTCTCTGGGAAATCGTGGAA - 3’ | 5’ - TCCAGTTTGGTAGCATCCATCA - 3’ |
| Socs3 | 5’ - ACCCTCCAGCATCTTTGTCG - 3’ | 5’ - GAACTCCCGAATGGGTCCAG - 3’ |
| Tnf | 5’ - AGCCCCCAGTCTGTATCCTT - 3’ | 5’ - CTCCCTTTGCAGAACTCAGG - 3’ |
| Wnt5a | 5’ - CAAATAGGCAGCCGAGAGAC - 3’ | 5’ - CTCTAGCGTCCACGAACTCC - 3’ |
Supplementary Table 1. Primers used for qRT-PCR.
